# Case report: A combined immunotherapy strategy as a promising therapy for MSI-H colorectal carcinomas with multiple HPD risk factors

**DOI:** 10.3389/fmed.2023.1051034

**Published:** 2023-05-04

**Authors:** Jinli Zhang, Lu Yang, Fanwei Kong, Di Wu, Baoru Hu, Jie Yang, Jiaxin He, Lei Liu

**Affiliations:** ^1^Department of Internal Medicine, Harbin Medical University Cancer Hospital, Harbin, China; ^2^The Genetic Analysis Department, YuceBio Technology Co., Ltd., Shenzhen, China; ^3^Department of Physical Diagnosis, Heilongjiang Province Hospital, Harbin, China

**Keywords:** colorectal carcinoma, MSI-H, immunotherapy, chemotherapy, HPD

## Abstract

Approximately 5% of advanced colorectal carcinomas (CRCs) and 12–15% of early CRCs are microsatellite instability-high (MSI-H) or mismatch repair-deficient (dMMR) tumors. Nowadays, PD-L1 inhibitors or combined CTLA4 inhibitors are the major strategies for advanced or metastatic MSI-H colorectal cancer, but some people still show drug resistance or progression. Combined immunotherapy has been shown to expand the benefit population in non-small-cell lung carcinoma (NSCLC), hepatocellular carcinoma (HCC), and other tumors while reducing the incidence of hyper-progression disease (HPD). Nevertheless, advanced CRC with MSI-H remains rare. In this article, we describe a case of an elder patient with MSI-H advanced CRC carrying MDM4 amplification and DNMT3A co-mutation who responded to sintilimab plus bevacizumab and chemotherapy as the first-line treatment without obvious immune-related toxicity. Our case provides a new treatment option for MSI-H CRC with multiple risk factors of HPD and highlights the importance of predictive biomarkers in personalized immunotherapy.

## Background

According to the latest GLOBOCAN data, the incidence and mortality of colorectal cancer (CRC) worldwide are 10 and 9.4%, respectively. In China, the incidence and mortality of male colorectal cancer are 23.25 and 17.28%, respectively, and the incidence and mortality of female colorectal cancer are 18.78 and 10.59%, respectively. Globally, it is estimated to be one of the cancers whose incidence is increasing, ranking second in mortality rate among all cancers (1). Although surgical resection remains an important treatment strategy for CRC, many researchers have now identified multiple biomarkers to guide personalized medical care approaches, including RAS family genes, BRAF, ERBB2, and MSI (2). Especially, immune checkpoint inhibitor (ICI) therapy could be an effective treatment option in advanced mismatch repair-deficient (dMMR)/microsatellite instability-high (MSI-H) CRC. Pembrolizumab, nivolumab alone, and nivolumab combined with ipilimumab have been approved by the Food and Drug Administration (FDA) for the treatment of these patients (3–5).

Immunotherapy has demonstrated efficacy in multiple cancer types; nevertheless, in some cases, the limitations of ICIs have inevitably emerged, including a low response rate, immune-related adverse events, primary and acquired resistance, and the rate of hyper-progression disease (HPD) ranging from 4 to 29% (6), which restrict its applicability in clinical practice. Some patients with tumor progression are treated with ICI, but the tumor deteriorates faster and progresses very rapidly, which is called HPD (7). HPD is associated with higher mortality and poor prognosis. There is evidence showing that some unique gene mutations and clinical characteristics, such as being older and the presence of multiple metastases, may be high-risk factors for HPD in different types of cancer (8). Therefore, it is necessary to pay attention to the risk factors in clinical practice to help patients timely to avoid HPD and make suitable treatment decisions. Several types of research studies have shown that combined immunotherapy is an effective strategy to avoid HPD, which can significantly improve the objective response rate and reduce the risk of HPD (9, 10). This combination therapy is widely used in non-small-cell lung cancer, advanced hepatocellular carcinoma, and other cancer types, while its efficacy and safety in CRC have not been reported, and information and experience are still limited (10–12).

Based on these, we focus on a case that combination immunotherapy overcomes caring for multiple HPD high-risk factors, which may provide a new strategy of immunotherapy for MSI-H colorectal cancer in precision medicine.

## Case presentation

**Figure 1:**
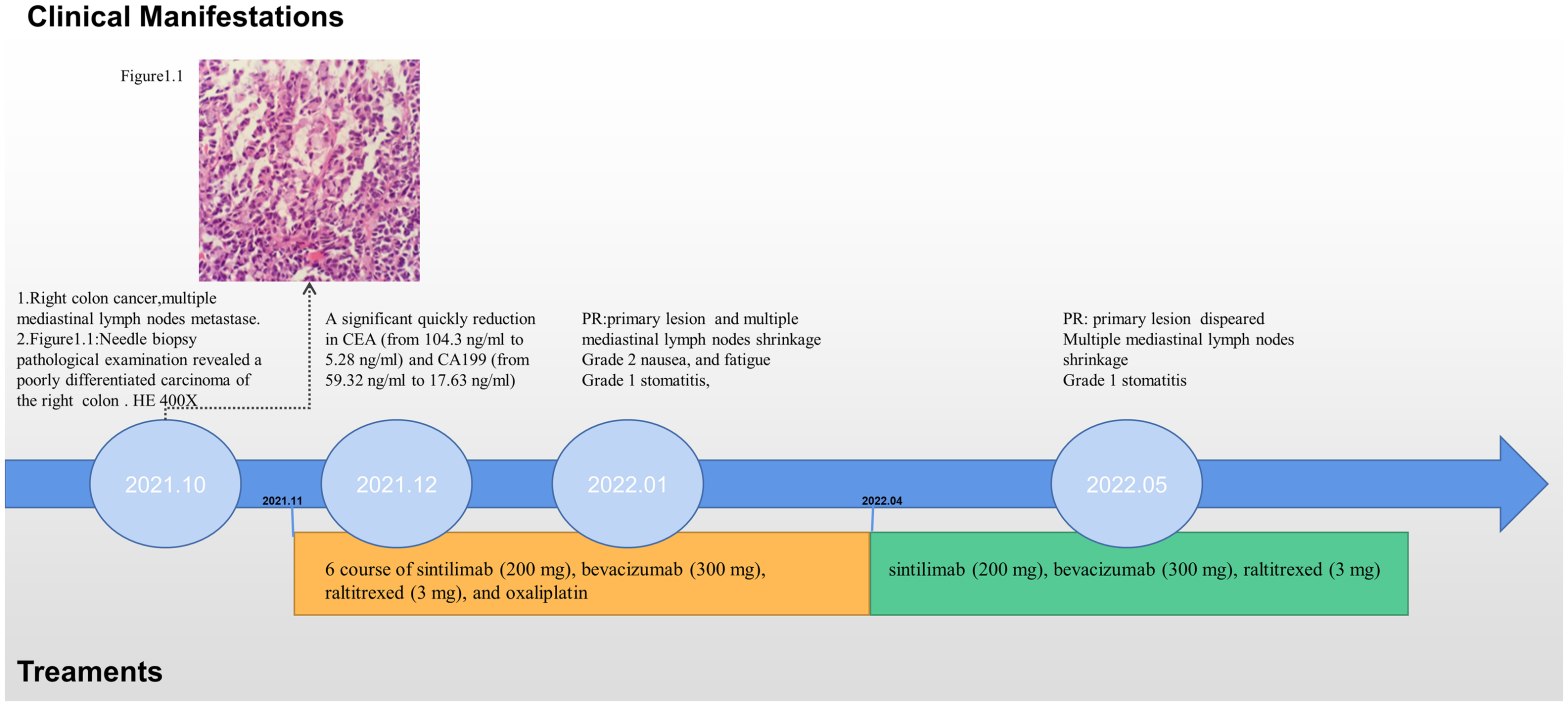
Schematic diagram showing the treatment record of the patient.

**Figure 2:**
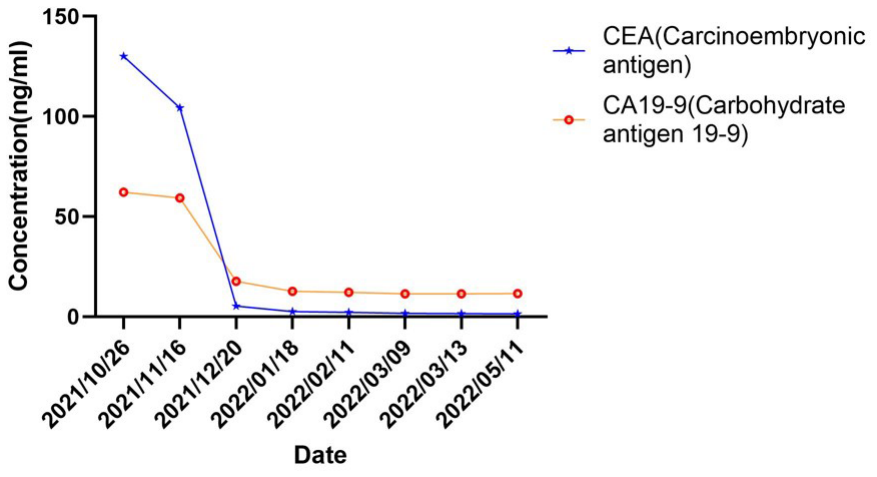
Serum tumor biomarker during treatment.

**Figure 3:**
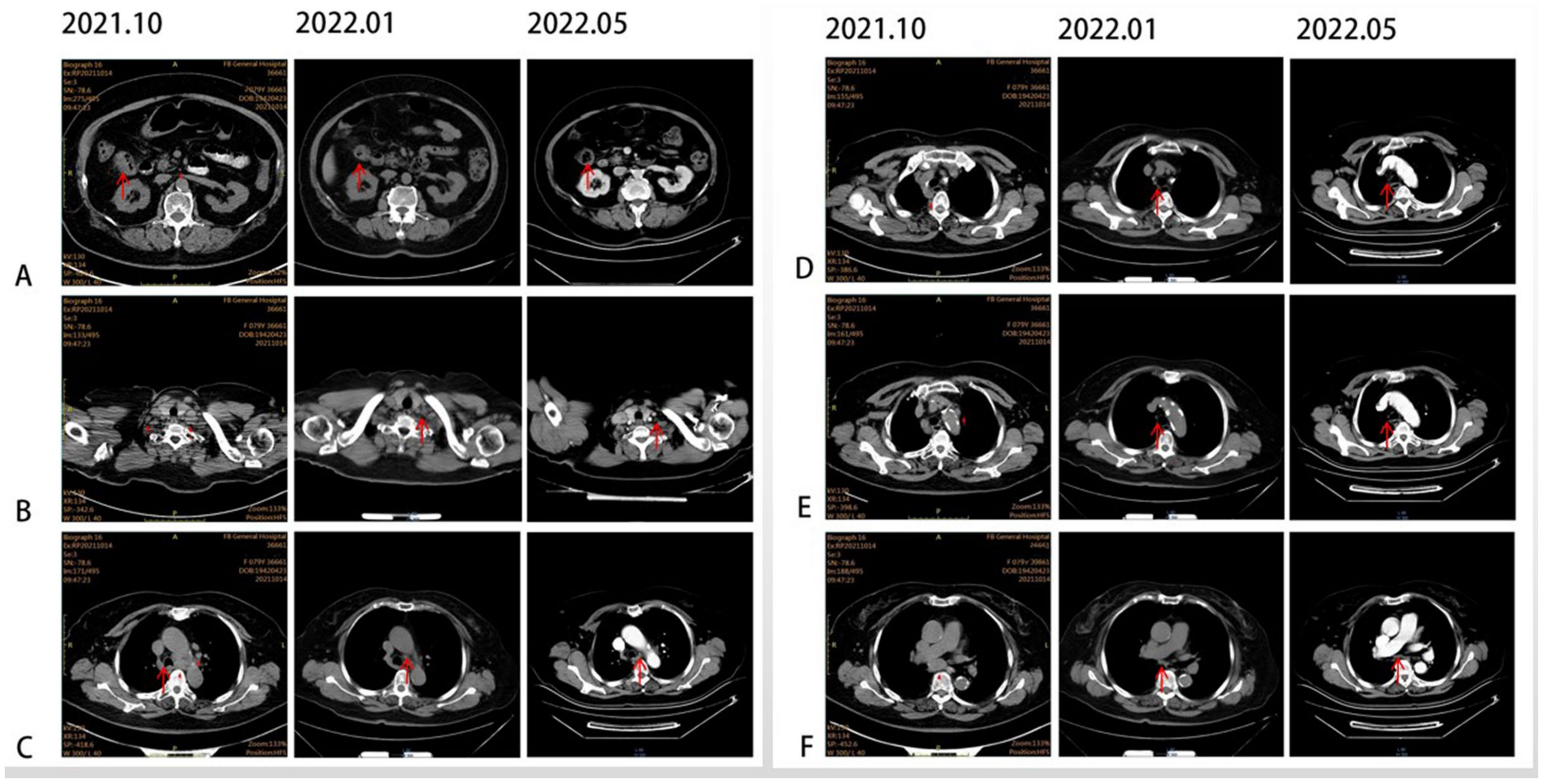
CT scans illustrating the changes in primary lesions and metastatic lymph nodes over time. **(A)** Primary lesion at the hepatic flexure (red arrow). **(B)** Supraclavicular lymph node (red arrow). **(C)** Tsubaortic lymph node (red arrow). **(D)** Paraesophageal lymph node (red arrow). **(E)** Retrotracheal lymph node (red arrow). **(F)** Subcarinal lymph node (red arrow).

In October 2021, an 80-year-old woman with a history of hypertension, diabetes mellitus, and coronary artery stenting was admitted to our hospital with an enlargement of a left-sided supraclavicular lymph node. She had presented with multiple mediastinal lymph node metastases by whole-body positron emission tomography–computed tomography (PET-CT). The patient was diagnosed with advanced Colon adenocarcinoma located in the right hepatic flexure hemicolon by pathological diagnosis (Figure 1.1). Next-generation sequencing (the customized panel covering 1,267 cancer-related genes in Yucebio Technology using MGISEQ platform) revealed MSI-H, TMB-H (89.82Mut/Mb), BRAF V600E, DNMT3A D686Tfs*19, and MDM4 amplification from the lymph node metastasis. See the Supplementary material for more details about the 1,267-gene. Immunohistochemical (IHC) staining was negative for PD-L1 (Dako 22C3). Considering that the patient was MSI-H carrying multiple risk factors related to HPD, and MEK inhibitor plus cetuximab or panitumumab is used for BRAF V600E-positive CRC in the non-first-line setting. She was provided first-line treatment with a four-drug combination strategy consisting of sintilimab (200 mg), bevacizumab (300 mg), raltitrexed (3 mg), and oxaliplatin (130 mg), and the chemotherapy dose was halved in November 2021. The treatment records are shown in Figure 1. After one cycle of treatment, the patient showed a significant quick reduction in CEA (from 104.3 to 5.28 ng/ml) and CA199 (from 59.32 to 17.63 ng/ml), indicative of response to the combination regime (Figure 2). After four cycles of treatment, the primary tumor mass reduced in size, the enlarged lymph nodes gradually shrank, and the CEA and CA199 returned to normal values, with a clinical evaluation of partial remission (PR) (Figures 2, 3A–F). In addition to stomatitis, nausea, and fatigue (grade 1–2 adverse events), there were no other adverse effects reported. The patient agreed with our recommendation to take sintilimab (200 mg) combined with bevacizumab (300 mg) and raltitrexed (3 mg) every 21 days as maintenance therapy from April 2022. In May 2022, a PET-CT scan showed that the primary tumor disappeared (Figures 3A–F). The patient remained in remission until the last follow-up in Mar 2023 with the adverse events disappearing, and she had full compliance due to the great clinical response.

## Discussion

Immune checkpoint has drastically changed the treatment landscape of patients with advanced MSI-H CRC (13). Compared with chemotherapy, single-agent immune checkpoint inhibitors as first-line have prolonged significantly disease-free survival in patients with MSI-H colorectal cancer; however, almost 40–60% of patients with MSI-H colorectal cancer still showed disease progression after receiving immune checkpoint inhibitor monotherapy (14). It also brings great challenges to the immunotherapy of advanced CRCs. Several cases have reported the phenomenon of HPD in MSI-H malignancy after receiving immunotherapy (15, 16). All of these suggest that the oncologist’s use of MSI as a sole biomarker may not be sufficient to determine whether patients receive immunotherapy. The case reports a patient with MSI-H CRC harboring multiple HPD risk factors who responded well to combined immunotherapy, providing a new option for CRC immunotherapy strategies and highlighting the importance of predictive biomarkers to immunotherapy.

The Chinese society of clinical oncology (CSCO) has formulated a guideline for high-throughput sequencing interpretation of CRC, recommending that the detection of several biomarkers related to immune HPD, such as MDM2/4, EGFR, and DNMT3A, should be considered to assist immunotherapy decisions (17). Unfortunately, our patient carries two of these gene variants: DNMT3A-inactivating variant D686Tfs*19 and MDM4 amplification 8.5. A previous research study has indicated that DNMT3A mutation (OR, 9.33; *p* = 0.03) and MDM2/4 amplification (OR: >11.9；*p* = 0.001) were closely associated with immunotherapy-related HPD (18). Recent large retrospective studies have shown that MDM2/4 amplification is a predictor biomarker and is significantly associated with lower survival with immune checkpoint inhibitors (19–21). Some studies attempted to investigate the mechanism of molecular biomarkers involving HPD in multiple tumor types, but it is not clear enough. Previous research showed that the DNMT3A-inactivating variant could regulate the immune microenvironment and resist M1 macrophage-killing effect *in vitro* and *in vivo*, which may escape the immune system (22). A mechanism inducing JAK–STAT signaling activation in tumor cells via IFN-γ levels increase has been suggested. This pathway further enhances IRF-8 expression, which might favor MDM2 expression and potentiate p53 inhibition, resulting in a dysregulation of cancer cell proliferation (23, 24). On the other hand, MDM2 amplification can induce resistance to ICI by reducing T-cell activation in malignancies (25, 26). Although none of these molecular alterations was found in CRC with HPD, which may be related to the low prevalence of MDM2/4 amplification (approximately 2%) and DNMT3A mutations (approximately 4%) in CRC. Suda et al. (27) demonstrated that the principle of rare MDM4 amplification in CRC is unclear, which is different from the mechanism of a mutually exclusive relationship between MDM2 alteration and TP53 inactivation (27). More importantly, our case carries MDM4 and DNMT3A co-mutation, which have not been reported in CRC by the Cbioportal Database.^1^

Multiple retrospective studies have shown that HPD patients are usually elder (aged ≥65 years old) (7). Notably, there is a lack of data on elderly patients (especially ≥75 years old). Previous studies have shown that older patients treated with ICIs have lower overall survival and PFS than younger patients and have an increased proportion of grade ≥3 immune-related adverse events (irAEs) (28, 29). Therefore, the tolerability of combined immunotherapy in elderly MSI-H CRC is worthy of attention. Other than that, three cases are reported of hyper-progression in MSI-H right Colon cancer (15, 16, 30), which suggests that right Colon cancer may be a high-risk factor for HPD.

Once HPD occurs, it usually causes irreversible damage and greatly reduces the survival time of patients. It is necessary to understand the risk factors of HPD and to avoid it in patients as much as possible. Sintilimab is a PD-1 inhibitor, which is approved for NSCLC, esophageal squamous carcinoma, and other malignant tumors by China National Medical Products Administration (NMPA) (31, 32). Research shows that chemotherapy combined with PD-1/PD-L1 inhibitors might suppress HPD, and cytotoxic drugs improve the immune microenvironment, such as the promotion of CD8+ T cell and NK cell infiltration and suppression of regulatory T cells (26). Meanwhile, chemotherapy can activate the antigen-presenting cell (APC)-mediated antigen presentation process, promoting an anti-tumor immune response to treat HPD (8). Anti-angiogenic therapy reduces the incidence of HPD upon that the increased VEGF induced by immune inhibitors promotes HPD via angiogenesis (8, 33, 34). Considering that our patient carries multiple HPD risk factors, including the elderly (80 years old), right colon cancer, multiple metastases, and MDM4 amplification and DNMT3A co-mutation, she received a four-drug combination strategy. The patient is receiving immunotherapy in combination with chemotherapy as maintenance therapy until now, achieving PR and great safety. Of course, longer follow-up is needed to demonstrate the efficacy. Additionally, the phase II/III clinical trials (NCT02997228, NCT04301557) focusing on immune-combination therapy efficacy in advanced MSI-H CRC as first-line therapy are currently in the recruiting phase. These results will be expected to support our conclusions in the future.

In conclusion, this is a highly clinically significant case report, which presents a case of an elderly MSI-H patient who overcame various HPD risk factors through a combination immunotherapy strategy. This case report may provide a new perspective to guide a personalized medical approach to CRC. This indicated that the comprehensive evaluation of different biomarkers is essential to identify the risk for immune HPD or resistance, especially in elderly patients, and contributes to the establishment of more accurate comprehensive personalized medical strategies. Meanwhile, the safety and efficacy of ICIs combination therapy in elderly MSI-H CRC was demonstrated.

## Ethics statement

Written informed consent was obtained from the individual(s) for the publication of any potentially identifiable images or data included in this article.

## Author Contributions

LL: conceptualization, methodology, and review. LY, JZ, and LL: data collection and analysis, and writing and editing. LY, JZ, and FK: modification. DW, JH, JY, and BH: literature research. All authors contributed to the article and approved the submitted version.

## Conflict of Interest

LY, JY, and JH are employed by YuceBio Technology Co. Ltd.

The remaining authors declare that the research was conducted in the absence of any commercial or financial relationships that could be construed as a potential conflict of interest.

## Publisher’s note

All claims expressed in this article are solely those of the authors and do not necessarily represent those of their affiliated organizations, or those of the publisher, the editors and the reviewers. Any product that may be evaluated in this article, or claim that may be made by its manufacturer, is not guaranteed or endorsed by the publisher.

## Footnotes

^1^ https://www.cbioportal.org/

## Supplementary material

The Supplementary material for this article can be found online at: https: //www.frontiersin.org/articles/10.3389/fmed.2023.1051034/full#supplementary-material
